# Current Understanding of CHIP’s Immunobiological Footprint with A Focus on Gastrointestinal Disorders: A Review of the Literature

**DOI:** 10.1007/s11912-026-01795-2

**Published:** 2026-06-10

**Authors:** Kirti Arora, Rishi Chowdhary, Rachel McNulty, Rahul Chowdhary, Wesam Aleyadeh, Megh Patel, Abhay Singh

**Affiliations:** 1 Cleveland Clinic Akron General, Akron, OH 44307 USA; 2 MetroHealth Medical Center, Cleveland, OH USA; 3 Cleveland Clinic, Cleveland, OH USA; 4 Liver Disease Branch, National Institute of Diabetes and Digestive and Kidney Diseases, National Institute of Health, Bethesda, MD USA; 5 B.J. Medical College and Hospital, Ahmedabad, Gujarat India; 6 Taussig Cancer Center, Cleveland, OH USA

**Keywords:** Clonal Hematopoiesis, Inflammation, Metabolic Dysfunction-Associated Steatotic Liver Disease, Inflammatory Bowel Disease, Colorectal Cancer

## Abstract

**Purposeof Review:**

Clonal hematopoiesis of indeterminate potential (CHIP) is characterized by the age-associated expansion of hematopoietic stem and progenitor cells (HSPCs) bearing somatic mutations in genes such as DNMT3A, TET2, ASXL1, and JAK2, without overt cytopenias or hematologic malignancy. CHIP is increasingly understood as a systemic inflammatory condition that influences extra-hematopoietic organs. Recent findings highlight its role in gastrointestinal (GI) pathology through immune dysregulation, chronic inflammation, and fibrogenic remodeling.

**Recent Findings:**

In the liver, CHIP, particularly TET2 and DNMT3A mutations, enhances IL-6 and NLRP3 inflammasome signaling in Kupffer cells, promoting hepatic inflammation, fibrosis, and heightened risk for cirrhosis and hepatocellular carcinoma. In inflammatory bowel disease (IBD), persistent cytokine exposure may select for pro-inflammatory clones, with TET2-mutant CHIP linked to severe disease phenotypes. In colorectal cancer, mutant macrophage and monocyte populations derived from CHIP alter tumor immune microenvironments, affecting tumor progression and therapeutic responsiveness, including checkpoint blockade efficacy.

**Summary:**

Together, these findings suggest that hematopoietic clones harboring specific mutations may act as upstream regulators of chronic inflammation and carcinogenesis within the GI tract. This review consolidates gene-specific mechanisms, interactions with the gut-liver immune axis, and implications for targeted interventions linking CHIP with gastrointestinal inflammation, fibrosis, and malignancy.

## Introduction

Clonal hematopoiesis of indeterminate potentialC (CHIP) describes the expansion of hematopoietic stem and progenitor cells bearing somatic mutations commonly associated with myeloid malignancies, in the absence of cytopenias or dysplastic features. This premalignant condition, an antecedent to myeloid malignancies such as MDS (Myelodysplastic Syndrome) and AML (Acute Myeloid Leukemia), was first formally defined in 2015 and is considered analogous to monoclonal gammopathy of undetermined significance (MGUS) within the myeloma spectrum. Both CHIP and MGUS are largely asymptomatic with an elevated risk for progression to overt malignancy [1]. The World Health Organization (WHO) defines CHIP as the presence of pathogenic mutations, typically in genes such as *DNMT3A, TET2, ASXL1, JAK2, *and *SF3B1*, at a variant allele fraction (VAF) ≥ 2%, in individuals without established hematologic disease or unexplained cytopenia [2]. These mutations lead to a proliferative advantage, usually through altered epigenetic regulation or resistance to inflammatory stress [3]. They are also associated with increased risk of hematologic neoplasms, cardiovascular disease, and all-cause mortality [4, 5].

CHIP is identified using Next-Generation Sequencing (NGS) platforms, including Whole Genome Sequencing (WGS), Whole Exome Sequencing (WES), and targeted panels. WES captures most pathogenic variants relevant for research, but targeted panels are more applicable to clinical diagnostics [6]. Additionally, with liquid biopsy platforms (cfDNA, ctDNA) often identifying CHIP mutations incidentally, the observed incidence, prevalence, and awareness of CHIP will continue to climb as these tests become widely used [7].

CHIP has recently been identified as a systemic modifier of inflammation and immune regulation. CHIP-associated mutations are linked to elevated levels of IL-6 and other proinflammatory cytokines, reflecting altered myeloid cell phenotypes that can potentiate local and systemic inflammatory responses [8, 9]. In experimental models, IL-6 signaling has been shown to propagate CHIP clonal expansion in the setting of bone marrow microenvironment (BME) changes associated with aging, obesity, and metabolic dysfunction [10, 11]. Similarly, CHIP has been mechanistically linked to a growing list of non-hematologic conditions including coronary artery disease, ischemic stroke, atherosclerosis, chronic liver disease, and autoimmune syndromes such as vasculitis and arthritis [12–19]. The proinflammatory macrophage-driven state induced by CHIP mutations, especially in *DNMT3A* and *TET2*, may amplify disease severity or affect therapeutic response [20–22].

This further highlights the evolving concept of CHIP as more than a pre-malignant lesion. The long latency period and high prevalence of CHIP in aging and at-risk populations for cancer make it a unique model of acquired somatic mosaicism with system-wide relevance [23]. Ongoing work aims to define how CHIP clones interact with local immune microenvironments, contribute to fibrogenesis, and shape neoplastic or pathogenic evolution across non-hematologic organ systems. This review will focus on the role of CHIP in inflammation-driven gastrointestinal conditions, particularly liver fibrosis, inflammatory bowel disease, and colorectal cancer.

### CHIP and Chronic Liver Disease

Metabolic-dysfunction-associated steatotic liver disease (MASLD) has led to increasing global health burden as it is projected to affect 27 million people in the United States by 2030 [24]. It develops following persistent hepatic injury, inflammation, and fibrogenesis [25]. Genetic contributors in hepatic lipid metabolism and lipotoxicity are increasingly recognized as central to MASLD progression, from lipid accumulation to severe scarring [26]. Mutations in the *PNPLA* gene are associated with hepatic steatosis, whereas *ATG7 *mutations increase the risk of hepatic cirrhosis and hepatocellular carcinoma (HCC) [27, 28]. Recent genomic analysis characterizes a phenomenon parallel to clonal hematopoiesis, where somatic mutations in metabolic genes like *FOXO1* and *CIDEB* accumulate in chronic liver disease. These mutations render hepatocytes resistant to insulin, protecting them from lipotoxicity and driving clonal expansion. While this adaptation ensures cell survival in lipid laden hepatocytes, it worsens metabolic dysfunction in MASLD [29].

Recent studies have implicated CHIP as a novel contributor to the pathophysiology of chronic liver disease [30]. In a large population-based analysis, individuals with CHIP had nearly twice the odds of developing chronic liver disease (OR 2.01; variant allele frequency ≥ 10%; 95% CI, 1.46–2.79; p < 0.001) and increased risk of Metabolic dysfunction-Associated Steatohepatitis (MASH) (OR 1.87; 95% CI, 1.17–3.01; p = 0.008). These associations remained independent of smoking and alcohol use. Mechanistic studies using murine models demonstrated that mice transplanted with *TET2*-deficient hematopoietic cells developed hepatic lobular inflammation with lymphoid aggregates in the absence of steatosis. In contrast, inflammation was mitigated in *TET2/NLRP3* double knockouts highlighting the importance of NLRP3-mediated signaling in *TET2*-driven hepatic injury. Kupffer cell–derived IL-6 was also identified as a key inflammatory mediator, echoing prior findings linking CHIP to NLRP3 activation in atherogenesis. These data suggest that TET2-mutant clones promote hepatic inflammation and fibrosis through paracrine cytokine signaling rather than direct steatotic injury [13].

CHIP's role in hepatocarcinogenesis has also been studied [31, 32]. In a study comparing patients with MASLD-HCC and those without HCC, CHIP was associated with a two-fold increased risk of malignancy (OR 2.01; 95% CI, 1.30–3.15; p = 0.002), independent of age, diabetes, and cirrhosis [31]. This association was more pronounced in males with attenuation after adjusting for age [31, 33]. *TET2* mutations resulted in the highest relative risk, (OR 4.8; 95% CI, 1.6–17.0; p = 0.02) consistent with previous evidence [31]. In contrast, *DNMT3A* mutations showed no significant association with HCC and has been shown to be inversely correlated (OR 0.60; 95% CI, 0.27–1.22; p = 0.24) [31]. While *JAK2* mutations were strongly linked to liver disease in earlier studies (OR 17.65; 95% CI, 4.32–72.15; p < 0.001), they were rarely identified in MASLD-HCC cases [13, 31]. Together, these findings emphasize the gene-specific roles of CHIP variants in hepatocarcinogenesis.

CHIP has also been implicated in immune complications following liver transplantation [34, 35]. In a retrospective analysis of patients with GVHD, *DNMT3A* mutations were observed in five of seven cases with available sequencing data, compared to just one of six non-GVHD patients (p = 0.04) [35]. In contrast, findings from a study with patients receiving HSCT with donor CH show that DNMT3A-related clonal hematopoiesis does not worsen transplant outcomes and is linked to lower relapse and better survival, particularly in recipients not given post-transplant cyclophosphamide (PTCy). The study reported no consistent increase in GVHD, and immune changes suggested a possible graft-versus-tumor effect. Donor CH clones could persist long-term without causing donor-cell leukemia when limited to *DNMT3A* or *TET2* mutations [36, 37].

Available data are limited but suggest that *DNMT3A*-related clonal hematopoiesis may also be associated with a higher risk of GVHD after allogeneic HSCT. On a similar note, it has been proposed that a potentially enhanced graft-versus-leukemia effect in this setting could improve overall outcomes. Data from liver transplantation show a similar pattern, with *DNMT3A*-mutant clones enriched among patients who develop severe GVHD-like immune complications. These similarities likely reflect the overlapping immunologic landscapes of HSCT and solid-organ transplantation and underlying clonal hematopoiesis. In HSCT, donor *DNMT3A*-mutant clones engraft into a fully reconstituted immune system where post-transplant cyclophosphamide limits excessive alloreactivity, allowing *DNMT3A*-driven myeloid inflammation to potentiate anti-leukemic effects without triggering GVHD. In liver transplantation, however, *DNMT3A*-CH arises in the host bone marrow and interacts with a partly intact, immunosuppressed immune environment, where heightened IL-6/NLRP3 signaling from mutant myeloid cells amplifies dysregulated inflammation and predisposes to GVHD, cytopenias, and hemophagocytic syndromes. Taken together, these findings indicate that the impact of CHIP on post-transplant immunity is highly context-dependent, shaped by mutation type, cellular origin (donor vs recipient), immune reconstitution dynamics, and the specific immunosuppressive regimen.

### CHIP and IBD

Clonal hematopoiesis has long been associated with aging and an increased risk of hematologic malignancies, but its relationship with chronic inflammatory conditions such as inflammatory bowel disease (IBD) has only recently been explored in detail [38–41]. Emerging evidence suggests a bidirectional pathogenic axis between IBD and CHIP, wherein chronic intestinal inflammation may accelerate clonal hematopoietic expansion, while CHIP-related systemic inflammatory signaling may conversely predispose to the onset or worsening of IBD.

A large study by Selvan et al. found that age and treatment both had specific selective pressures on clonal populations. Individuals over the age of 45 years exhibit notably higher rates of myeloid (M-CHIP) in ulcerative colitis (UC) and lymphoid (L-CHIP) clones are seen in older IBD patients overall [42]. Younger Crohn’s disease (CD) patients were frequently seen to carry TET2 mutations, correlating with more severe inflammatory outcomes and heightened hematologic malignancy risk [42]. Steroid use was associated with increased CHIP (P = 0.05), while anti-TNF therapy was associated with decreased myeloid-CHIP (P = 0.03). Other studies have also shown increased risk of IBD in patients with CHIP, especially with mutations in *JAK2, ASXL1* and *DNMT3A,* large CHIP clones and selective evolution of clonal population in this patient population [42].

Further investigations assessed whether chronic IBD-related inflammation accelerates malignant transformation. Earlier studies have reported higher risk of lymphoid malignancies within the first year of diagnosis, myeloid neoplasms (MN) overall and myelodysplastic syndrome (MDS) transplantation [43, 44]. With the widespread use of NGS for mutational profiling, it has been possible to look into CHIP and specific mutations to ascertain a biological mechanism for this increased risk. In an analysis done by Cumbo et al., 85% of the patients with IBD and associated hematologic malignancies were found to have CH mutations, most commonly *DNMT3A*. In several cases, individuals carried multiple driver mutations and showed dominant clonal populations with variant allele frequencies greater than 10%, suggesting that chronic inflammation may create a permissive environment that supports clonal expansion and increases the potential for malignant progression. This high prevalence may warrant frequent screening of these patients for associated myeloid malignancies [45].

Subsequent retrospective analyses have also revealed that there are early subtle hematologic disturbances in IBD patients preceding MN diagnoses, leading to MDS, challenging prior assumptions about cytopenias predominantly being microcytic and reactive [46–49]. Collectively, these findings suggest chronic intestinal inflammation in IBD reshapes hematopoietic clonal dynamics, selecting and expanding specific mutations leading to elevated risk of hematologic malignancy at younger ages, and increased severity of IBD.

### Association between Clonal Hematopoiesis and Colorectal Cancer

Different clinical and preclinical studies support a strong association between CH and CRC [50, 51]. Early mechanistic evidence came from a mouse model of colitis-associated colon cancer driven by *DNMT3A*-mutated hematopoiesis; animals were seen to have a higher risk of development of adenocarcinoma, disease severity and tumor burden. These changes were accompanied by enhanced intratumoral angiogenesis that likely led to CRC progression, with response to the angiogenesis inhibitor axitinib, highlighting a potential therapeutic strategy for CH-driven CRC [52]^.^ A large prospective study by Liu et al. demonstrated a 20% increase in risk of CRC in patients with CHIP (p 0.006), especially in females and individuals over the age of 60 years with *TET2* and *ATM* gene mutations [53]. Another study by Desai et al. did not find an increase in the incidence of CRC in female patients with CHIP, where an increased risk of mortality was seen [54]. Findings from both these studies highlight the significant role of CHIP in disease and mortality risk, advocating for its role as a risk stratification tool.

CHIP is also found in various solid tumors, known as Tumor Infiltrating clonal hematopoiesis (TICH), which is emerging as an independent risk factor for disease recurrence and mortality [55–58]. CHIP mutations shape the tumor-immune interface by altering both the tumor microenvironment (TME) and systemic immune dynamics [59–61]. *TET2 *and* DNMT3A* mutations have been identified in immune cells within the TME and exert divergent effects depending on context by promoting or restraining tumor growth. In solid tumors treated with Immune Checkpoint Inhibitor therapy (ICI), CHIP may enhance anti-tumor immunity, whereas in the absence of ICI use, it may sustain the immunosuppressive function to promote tumorigenesis [20, 62, 63]. The role of CHIP in risk stratification extends beyond disease progression and therapeutic response to severity of adverse events, such as Cytokine Release Syndrome (CRS) after Chimeric Antigen Receptor T cell (CAR-T) therapy [64].

Conversely, results from a prospective analysis for CH in patients from the FIRE 3 trial, which investigated the use of FOLFIRI with cetuximab vs bevacizumab in metastatic CRC (mCRC) had contrasting findings. Although there was a significant presence of CH in 36% of patients with mCRC, most commonly involving *DNMT3A, TET2, PPM1D,* and *ASXL1* mutations in older individuals with prior chemotherapy exposure, there was seen improved overall survival (OS), without any difference in progression-free survival (PFS). CH-positive patients had higher rates of grade 3 to 4 diarrhea and thromboembolic events [65]. These findings may be due to the CHIP-mediated immune environment for tumor control and potential enhancement of response to further lines of therapy, suggesting importance of cancer type and therapy in determining CHIP’s role in prognostication.

**Fig. 1:**
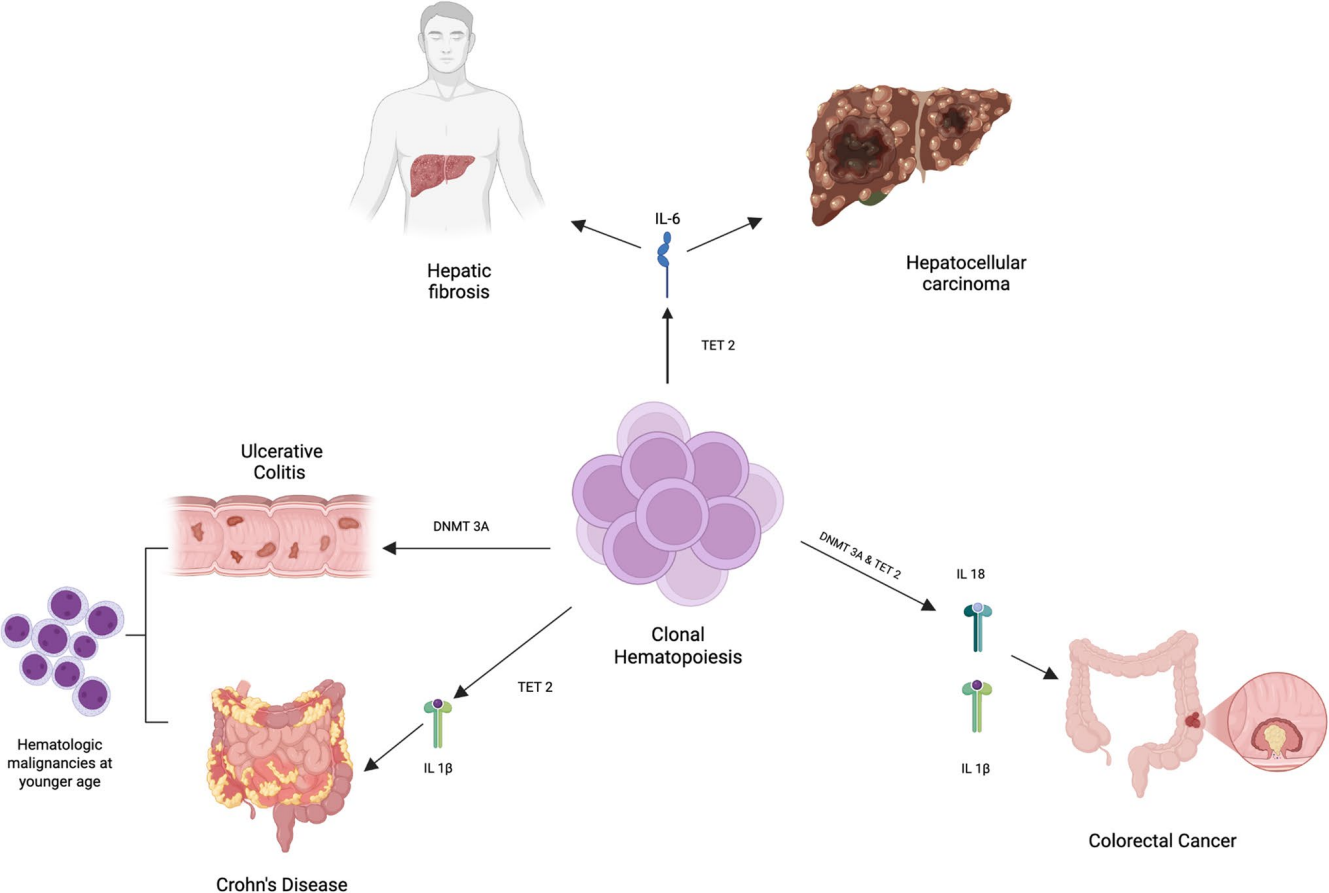
Associations of Clonal Hematopoiesis with GI pathology. Created with Biorender.com

CHIP has also been associated with other GI cancers, like esophagoduodenal showing worse OS and pancreatic showing improved PFS in those treated with ICI compared to worse OS in those treated with conventional chemotherapy [51, 66]. Detection of CHIP may warrant increased surveillance for adverse events as seen in CRC. This highlights the need for further prospective studies to determine the prognostic significance of CH in specific tumor contexts. A deeper understanding of how specific CHIP mutations influence immune tone, whether proinflammatory or immunosuppressive, will be essential for developing risk stratification tools and targeted therapies for CRC and other solid tumors. (Fig. 1).

### CHIP and Conditions Having GI Manifestations

Outside of primary GI pathology, CHIP is also seen in conditions presenting with GI symptoms. VEXAS syndrome is a severe adult-onset inflammatory disease caused by somatic mutations in *UBA1* in myeloid precursors. Loss of functional *UBA1* disrupts the ubiquitin-activating enzyme E1 pathway resulting in impaired protein degradation, activation of the unfolded protein response, and chronic innate immune signaling [67, 68]. Clinically, VEXAS presents with systemic inflammation, cytopenias, chondritis, vasculitis, and skin findings. Gastrointestinal symptoms occur in some patients and usually manifest as abdominal pain, diarrhea, and bleeding related to vasculitis or granulomatous bowel involvement. *TET2* and *DNMT3A* mutations are frequently co-detected in affected individuals [69]. Conventional immunosuppressive therapy may be ineffective in these patients because mutation-driven, cell-intrinsic activation of the myeloid clone sustains inflammation despite broad dampening of immune cells. Thus, allogeneic hematopoietic stem cell transplantation is currently being explored as a potential curative approach [68].

These syndromes reflect the capacity of CHIP-related mutations to induce chronic inflammation in the gastrointestinal tract independent of inherited immunodeficiencies. Recognizing these presentations is critical for early diagnosis and consideration of mutation-directed therapies in patients with atypical or treatment-resistant GI inflammation.

## Conclusion

CHIP has emerged as a clinically relevant phenomenon beyond its role as a precursor to hematologic malignancies. Its presence influences a spectrum of gastrointestinal diseases through mechanisms rooted in immune dysregulation, cytokine signaling, and chronic inflammation. Somatic mutations in genes such as *TET2, DNMT3A, JAK2*, and *PPM1D *shape inflammatory responses and impact disease phenotypes across diverse GI pathologies, including steatotic liver disease, inflammatory bowel disease, colorectal cancer, and CHIP-associated inflammatory syndromes like VEXAS (Table 1). Accumulating evidence suggests that chronic inflammation promotes clonal expansion and may accelerate malignant transformation in susceptible hosts. These findings highlight a bidirectional relationship between CHIP and inflammatory GI disorders that has important implications for risk stratification, surveillance, and potential therapeutic targeting.

**Table 1:** Epidemiologic associations between CHIP and inflammation-driven gastrointestinal conditions

Domain	Study (First author, year)	Design & Population (N)	CHIP Definition (assay, genes, VAF)	Outcome	Effect size (OR/HR, 95% CI, p)	Covariate Adjustment	Gene-Specific Signals	Inflammation Markers (IL-6, hsCRP)	Notes/Comments
Liver [32]	Marchetti A, 2024	Case–control (NASH, fibrosis, HCC)	WES; CHIP genes (TET2, TP53, ASXL1, etc.)	Prevalent HCC	OR 3.04 (p < 0.01)	Adjusted for age, BMI, diabetes	TET2 driver in HCC	IL-6, IL-1β elevated	Strong NASH–CHIP–HCC correlation; fibrosis subgroup validated
IBD [42]	Selvan ME, 2024	Cross-sectional (IBD vs controls, n = 441)	WES; canonical CHIP genes	Prevalent CHIP	OR 2.3 (p < 0.03)	Logistic regression (age, sex, treatment)	TET2, DNMT3A enriched	IL-6 pathway overlap with IBD	Distinct CHIP profile in IBD; pathway overlap noted
Colorectal [53]	Liu Y, 2025	Prospective matched cohort (UK Biobank)	Deep sequencing (WES, VAF ≥ 2%); genes: TET2, DNMT3A, ASXL1, JAK2	Incident CRC	OR 1.20 (p = 0.006); stronger for TET2, DNMT3A	Adjusted for age, sex, BMI, lifestyle	TET2, DNMT3A significant	No consistent IL-6/CRP association	Large UK dataset, well-powered; gene-specific results highlighted
Pancreatic [66]	Krishnan T, 2025	Review synthesis of CHIP in solid tumors	Summarized across assays (targeted panels, VAF ≥ 2%)	Incident & prevalent cancer	Directional links noted	Adjusted for smoking, age, stage	TET2, DNMT3A common	IL-1β, IL-6 pathways	Good synthesis linking CHIP to inflammation & tumor growth

Abbreviations: ASCVD – Atherosclerotic cardiovascular disease; DNA – Deoxyribonucleic acid; FHS – Framingham Heart Study; HCC – Hepatocellular carcinoma; hsCRP – High-sensitivity C-reactive protein; IL – Interleukin; MASH – Metabolic dysfunction–associated steatohepatitis; NASH – Nonalcoholic steatohepatitis; TP53 – Tumor protein p53; VAF – Variant allele frequency; WES – Whole-exome sequencing; WGS – Whole-genome sequencing.

Prospective, mutation-specific interventions are being explored in clonal hematopoiesis with different therapeutic strategies tailored to underlying genetic alterations. *JAK2*-CH may respond to *JAK* inhibitors, while *IDH1/2-*CH can be targeted with IDH inhibitors [70, 71].

Emerging data in *IDH1/2*-mutant CH/CCUS suggest that *IDH1* inhibition (e.g., ivosidenib) may not only improve hematologic parameters but also reduce systemic inflammation (including normalization of elevated hsCRP and symptomatic improvement in inflammatory manifestations) [72]. Additionally, *TET2*-CH has shown potential benefit from TOP1-targeted drugs (irinotecan, topotecan), and *PARP1* inhibitors (olaparib)[73]. *DNMT3A* pathways are targeted by PI3Kα/δ inhibitors and DOT1L drugs [74–76]. In addition, emerging metabolic anti-inflammatory agents, including GLP-1 receptor agonists and statins, are also being considered for long-term disease risk modification [77, 78] (Table 2). Future prospective studies are needed to identify reliable biomarkers that enable early detection of clonal hematopoiesis. And evaluate targeted strategies to prevent progression from stable CH to overt malignancy.

**Table 2:** Future directions: Mutation-stratified interventions for CHIP in inflammation-driven GI disease:

Mutation/Pathway	Pathobiology	Candidate interventions	GI settings	Notes
JAK2 (inflammation/thrombosis axis) [70]	Hyperactive JAK/STAT signaling, cytokine priming	JAK inhibitors (e.g., ruxolitinib)	MASH/fibrosis; IBD with systemic inflammation; post-polypectomy CRC risk	Monitor cytopenias; thrombosis endpoints
TET2 (inflammasome/IL-1 axis) [73]	Myeloid hyper-IL-1/IL-6 signaling	TOP1-targeted drugs (irinotecan, topotecan); PARP1 inhibitors (olaparib)	MASH → fibrosis; colitis activity	Rationale strongest in TET2-CH
DNMT3A (myeloid priming/metabolic inflammation) [74–76]	Epigenetic skewing, low-grade inflammation	PI3Kα/δ inhibitors; DOT1L; weight-loss/metabolic therapy (metformin)	MASH; post-CRC resection surveillance	Broad CH-applicability; pragmatic designs feasible
IDH1/2 (oncometabolite signaling) [72]	2-HG–driven epigenetic dysregulation	IDH1/2 inhibitors (Ivosidenib/Olutasidenib/Enasidenib)	High-risk MASH/fibrosis with IDH-CH	Niche and rare population
Pan-CH (any gene) risk-reduction [13, 77, 78]	Clone-associated systemic inflammation	Anti-inflammatory (IL-1/IL-6), statins (simvastatin), lifestyle	Across liver fibrosis, IBD, CRC prevention	↑ Risk of chronic liver disease (OR 2.01) and MASH (OR 1.87)

Abbreviations: JAK – Janus kinase; STAT – Signal transducer and activator of transcription; hsCRP – High-sensitivity C-reactive protein; VAF – Variant allele frequency; MASH – Metabolic dysfunction–associated steatohepatitis; NLRP3 – NOD-like receptor family pyrin domain containing 3; TNFα – Tumor necrosis factor alpha; IDH – Isocitrate dehydrogenase; 2-HG – 2-hydroxyglutarate; GLP-1 – Glucagon-like peptide-1; RA – Receptor agonist; SGLT2i – Sodium–glucose cotransporter-2 inhibitor; ALT – Alanine aminotransferase; AST – Aspartate aminotransferase; CH – Clonal hematopoiesis.

## Key References

- Lee YT, Tseng HR, Yang JD. Clonal hematopoiesis of indeterminate potential and risk of hepatocellular carcinoma: New kids on the block. *Hepatol Baltim Md*. 2024;80(4):763–765. 10.1097/HEP.0000000000000898

o This article highlights emerging evidence linking CHIP to an increased risk of HCC, emphasizing CHIP as a novel systemic driver of inflammation-mediated hepatocarcinogenesis in metabolic liver disease.

- Liu Y, Xi Z, Zhou J, et al. Clonal Hematopoiesis of Indeterminate Potential as a Predictor of Colorectal Cancer Risk: Insights from the UK Biobank Cohort. *Cancer Epidemiol Biomark Prev Publ Am Assoc Cancer Res Cosponsored Am Soc Prev Oncol*. 2025;34(3):405–411. 10.1158/1055-9965.EPI-24-1342

o This large UK Biobank cohort study demonstrates that CHIP is associated with an increased risk of colorectal cancer, providing important population-level evidence supporting CHIP as a potential biomarker for cancer susceptibility and risk stratification in gastrointestinal malignancies.

- Cumbo C, Tarantini F, Zagaria A, et al. Clonal Hematopoiesis at the Crossroads of Inflammatory Bowel Diseases and Hematological Malignancies: A Biological Link? *Front Oncol*. 2022;12:873896. 10.3389/fonc.2022.873896

o This study explores the relationship between clonal hematopoiesis and IBD, highlighting how chronic intestinal inflammation may promote clonal expansion and increase susceptibility to hematologic malignancies.

## Data Availability

No datasets were generated or analysed during the current study.
